# The impact of the COVID-19 pandemic on the mental health and academic performance of medical postgraduates

**DOI:** 10.3389/fpubh.2022.948710

**Published:** 2022-09-15

**Authors:** Yibo Yu, Qiaomei Tang, Haifei Shi, Ting Chen, Yaping Wang, Hanguang Hu, Ke Yao

**Affiliations:** ^1^Eye Center, The Second Affiliated Hospital, Zhejiang University School of Medicine, Hangzhou, China; ^2^Zhejiang Provincial Key Lab of Ophthalmology, Hangzhou, China; ^3^Department of Orthopedics, First Affiliated Hospital, Zhejiang University School of Medicine, Hangzhou, China; ^4^Key Laboratory of Cancer Prevention and Intervention, Ministry of Education, Cancer Institute, The Second Affiliated Hospital, Zhejiang University School of Medicine, Hangzhou, China; ^5^Department of Cardiology, The Second Affiliated Hospital, Zhejiang University School of Medicine, Hangzhou, China; ^6^Department of Medical Oncology, Key Laboratory of Cancer Prevention and Intervention, Ministry of Education, The Second Affiliated Hospital, Zhejiang University School of Medicine, Hangzhou, China

**Keywords:** COVID-19 pandemic, medical postgraduates, mental health, academic performance, education

## Abstract

**Background:**

The Coronavirus disease 2019 (COVID-19) has presented a major challenge to the health, economic, and social sectors of the entire world. This study aimed to investigate the mental health and academic performance of medical postgraduates during the COVID-19 pandemic in China.

**Methods:**

A cross-sectional online survey was conducted from March 20 to April 20, 2022 at the Zhejiang University School of Medicine in China. The questionnaire consisted of three parts: general information, mental health and academic performance. Mental health outcomes were assessed according to the Generalized Anxiety Scale (GAD-7) and the Patient Health Questionnaire-9 Scale (PHQ-9).

**Results:**

A total of 153 valid questionnaires were obtained. Of the medical postgraduates in this study, (1) 41.8% had no anxiety symptoms. In addition, 51.0% had mild anxiety symptoms during the COVID-19 pandemic. None of the participants had a severe anxiety or depression disorder; (2) Females experienced significantly more symptoms in mental health measure scores than the males (*P* < 0.01); (3) 78.4% believed that the COVID-19 pandemic had varying degrees of impact on their academics. Doctoral postgraduates showed greater academic stress, and they were more worried about not meeting graduation standards than the master's postgraduates (*P* < 0.05). There were no significant differences between the surgical postgraduates and internal postgraduates in either mental health or academic performance.

**Conclusions:**

Our study found that the COVID-19 pandemic has had a mild impact on the mental health and academic performance of medical postgraduates in China. Females experienced significantly more symptoms on mental health measure scores than the males. Doctoral postgraduates showed greater academic stress than the master's postgraduates. There is uncertainty regarding how long this COVID-19 situation will persist and increasing recognition that there may be periods of recurrence in the future. We need more active curricular innovation and transformation to maintain and improve medical postgraduates' mental health and academic performance.

## Introduction

Since the World Health Organization (WHO) declared a global emergency on January 30, 2020 concerning the novel coronavirus disease-19 (COVID-19) outbreak (1, 2), COVID-19 has presented a huge challenge to the health, economic and social sectors of the entire world. The WHO reported 514 million confirmed cases of COVID-19 and 6.2 million confirmed deaths worldwide as of May 4, 2022 (3). Due to COVID-19, mental health illnesses including anxiety, depression, stress, and sleep disorder have become prevalent problems across the world. Various studies demonstrated that during the COVID-19 pandemic, mental health problems such as fear, anxiety and depression were common among the general public, patients, medical staff, children, and older adults (4–8). Previous research concluded that throughout 2020, the COVID-19 pandemic led to a 27.6% increase in cases of major depressive disorders and 25.6% increase in cases of anxiety disorders globally (9). Among general population in China, the prevalence of depressive symptoms, anxiety, and stress ranging from moderate to severe were, respectively, 16.5, 28.8, and 8.1% (10).

Considered as a top-down management system, the Chinese government has effectively adopted a dynamic clearance policy to control the COVID-19 pandemic over the past 2 years (11). Specifically, the local government is carrying out community, block, district, or regional lockdowns as needed whenever COVID-19 cases are reported. Therefore, schools and public places are closed in lockdown areas, and students must study online at home. For postgraduate medical students, many courses have been transferred from classroom learning to online learning due to the changes in learning and living environments. Consequently, medical students are unable to communicate effectively with teachers, and there is a lack of internship opportunities for clinical learning in the hospital (12). These unprecedented changes and unique challenges have affected medical students' mental health and academic performance. There are a growing number of studies on how college and medical students have adapted to the COVID-19 pandemic (13–16). Mental health disorders among Greek medical students have been reported in connection with the COVID-19 pandemic (17). Similar findings have also been reported in China. Specifically, compared with non-medical students, medical students had fewer mental health symptoms in the early stages of the COVID-19 pandemic since they are more knowledgeable about COVID-19 (18).

Medical students in China have experienced various restrictions and challenges due to the prevention and control of the pandemic during the past 2 years. Compared with the beginning of the pandemic, medical students have become more and more adaptable to these various restrictions and challenges, resulting in our study of the impacts on their mental health and academic performance. Undergraduate medical students in China mainly study in the classroom. Meanwhile, postgraduate medical students need to spend a large amount of time in the hospital for clinical learning and research under their supervisors' hands-on guidance. Therefore, the COVID-19 pandemic has had a greater impact on postgraduate medical students' academic studies and clinical learning in the hospital. Therefore, the aim of the present study is to evaluate the impact of the 2 year COVID-19 pandemic on the postgraduate medical students' mental health (e.g., anxiety, depression) and academic performance in China. The present study was a web-based, cross-sectional study. The questionnaire consisted of three parts: general information, mental health and academic performance.

## Methods

### Setting and sample

A cross-sectional survey using structured questionnaires was conducted among medical postgraduate students at the Zhejiang University School of Medicine in Hangzhou, China. The data were collected from March 20 to April 20, 2022. During this period, students in China were still affected by the COVID-19 pandemic. The inclusion criteria were as follows: students (1) had normal mental state in the school; (2) who agreed to participate in the study after being fully informed of details of the study. Exclusion criteria were any physical or neurological disease, substance use, psychotic disorders, and personality disorders. We adopted the method of stratified random sampling, and divided the postgraduates in the school of Zhejiang University School of Medicine into a doctoral class of surgery, a master's class of surgery, a doctoral class of internal medicine and a master's class of internal medicine. Forty to fifty postgraduate students were randomly selected from each class, and 177 medical postgraduate students were selected as research objects. They were invited to voluntarily participate in the survey through the network platform (http://www.wjx.cn, which is one of the most popular electronic survey platforms in China). We tested its internal consistency in a pilot study of 50 students. The questionnaire had a high level of internal consistency among our study population, with a Cronbach's alpha 0.88. An online survey questionnaire was distributed randomly to students *via* WeChat. Participants were asked to read the instructions about the purpose and methods to fill out the questionnaire carefully. Participants were also informed that the survey was anonymous. A total of 177 questionnaires were sent out. Twenty-four of the retrieved questionnaires were invalid and were thus excluded from the study, and 153 were valid. The effective survey questionnaire retrieval rate was thus 86.44%. Ethical approval was obtained from the ethics committee of the Second Affiliated Hospital, School of Medicine, Zhejiang University in China. All participants provided written informed consent prior to participating in the study without identifiable data.

### General information

The first part of the questionnaire asked about the basic demographic information of the participants (such as gender, home location, grade, academic major, marital status and health status).

### Mental health

The second part of the questionnaire addressed the participants' general anxiety and depression disorders during the COVID-19 pandemic. The questionnaire was adapted from the Generalized Anxiety Scale (GAD-7) and the Patient Health Questionnaire-9 Scale (PHQ-9) (19–21). The revised GAD-7 questionnaire had a total of 6 items. Example item is “I feel nervous or anxious during COVID-19 pandemic.” Items are scored on a four-point Likert scale ranging from 0 (never) to 3 (always), with scores of 0, 1, 2 and 3 according to “none at all,” “a few days,” “more than half of the days” and “almost every day.” According to the final score of the questionnaire, the research subjects were divided into the normal group (0–3 points) and the anxiety symptom group (3–18 points). The revised PHQ-9 questionnaire had a total of 4 items. The total score of the questionnaire is 0–12 points, 0–3 points were divided into normal group, and those with ≥3 points were judged to have depressive symptoms. The higher the score, the more severe the social anxiety or depression. In previous studies on Chinese general and adolescent populations, the GAD-7 and PHQ-9 scores showed good psychometric properties (22–24).

### Academic performance

The third part of the questionnaire was the investigation of the personal academic performance of the clinical medical postgraduates. They were asked about the overall impact on academics, worrying that they would not meet graduation standards, the percentage of offline courses converted to online courses, the percentage of practice opportunities reduced and the frequency of counseling and academic communication with graduate tutors.

### Statistical analysis

The data from the retrieved electronic survey questionnaires were gathered using Questionnaire Star. Categorical variables were described as counts (*n*) and percentages (%). The questionnaire scores were expressed as mean ± SD, and between-group statistical comparisons were made using an independent sample *t*-test or the Mann-Whitney *U* test, depending on normal distribution (25, 26). Two-sided *P*-values were considered significant at *p* < 0.05. All data were analyzed using SPSS version 22.0 software.

## Results

### Demographic Characteristics

A total of 153 medical postgraduates completed the survey. Table 1 shows the distribution of the study participants. Most (67.32%) were female. Those who completed the survey were 45.76% master's students and 54.24% doctoral students. 55.56% of the postgraduates who participated in this questionnaire were surgical students, and 44.44% were internal medical students. Most were unmarried (88.24%). The current health status of the postgraduates was basically good, and the proportion of students with a poor health status was 0%, based on self-assessment.

**Table 1:** Demographic characteristics of the study subjects.

**Essential information**	**Classification**	**No. of people**	**Percentage (%)**
Gender	Male	50	32.68%
	Female	103	67.32%
Home location	Town	93	60.78%
	Countryside	60	39.22%
Grade level	First year, master's program	31	20.26%
	Second year, master's program	20	13.07%
	Third year, master's program	19	12.42%
	First year, doctoral program	21	13.73%
	Second year, doctoral program	20	13.07%
	Third year, doctoral program	19	12.42%
	Fourth year, doctoral program	12	7.84%
	Fifth year, doctoral program	11	7.19%
Major	Surgical	85	55.56%
	Internal medicine	68	44.44%
Marital status	Unmarried	135	88.24%
	Married	18	11.76%
	Divorced or otherwise	0	0%
Current health status	Very good	70	45.75%
	Good	79	51.63%
	General	4	2.61%
	Poor	0	0%

### The impact of the COVID-19 pandemic on the mental health of medical postgraduates

Most of the medical postgraduates who participated in this study had no or mild mental health symptoms during the COVID-19 pandemic (Table 2). Most had low scores for anxiety or depression. Only two people (1.3%) felt that it was hard to relax in the COVID-19 environment almost every day. One person (0.7%) had a severe symptom of anxiety. In addition, none of the participants had a severe depression disorder.

**Table 2:** The impact of the COVID-19 pandemic on mental health of medical graduate studies.

**Questionnaire content**	**Absolutely not (0)** **[95% CI]**	**A few days (1)** **[95% CI]**	**More than half of the days (2)** **[95% CI]**	**Almost everyday (3)** **[95% CI]**
Feeling nervous or anxious during COVID-19 pandemic	64 (41.8%) [33.9–49.7%]	78 (51.0%) [43.0–59.0%]	11 (7.2%) [3.1–11.3%]	0 (0.0%) [0.0–0.0%]
Worrying cannot be stopped or controlled during COVID-19 pandemic	79 (51.6%) [43.6–59.6%]	64 (41.8%) [33.9–49.7%]	9 (5.9%) [2.1–9.7%]	1 (0.7%) [0.0–1.9%]
Are you worrying too much about various things because of the COVID-19 pandemic	65 (42.5%) [34.6–50.4%]	76 (49.7%) [33.9–49.7%]	12 (7.8%) [3.5–12.2%]	0 (0.0%) [0.0–0.0%]
It's hard to relax in a COVID-19 environment	91 (59.5%) [51.6–67.3%]	52 (34.0%) [26.4–41.6%]	8 (5.2%) [1.7–8.8%]	2 (1.3%) [0.0–3.1%]
Become easily irritable during COVID-19 pandemic	85 (55.6%) [47.6–63.5%]	59 (38.6%) [30.8–46.4%]	8 (5.2%) [1.7–8.8%]	1 (0.7%) [0.0–1.9%]
Fearing that something terrible is about to happen during COVID-19 pandemic	97 (63.4%) [55.7–71.1%]	49 (32.0%) [24.5–39.5%]	6 (3.9%) [0.8–7.0%]	1 (0.7%) [0.0–1.9%]
Not feeling motivated or having fun doing things during COVID-19 pandemic	99 (64.7%) [57.0–72.4%]	42 (27.5%) [20.3–34.6%]	12 (7.8%) [3.5–12.2%]	0 (0.0%) [0.0–0.0%]
Feeling down, depressed or hopeless due to COVID-19 pandemic	91 (59.5%) [51.6–67.3%]	55 (35.9%) [28.3–43.6%]	7 (4.6%) [1.2–7.9%]	0 (0.0%) [0.0–0.0%]
Difficulty falling asleep, restless sleep or more sleep during COVID-19 pandemic	109 (71.2%) [64.0–78.5%]	39 (25.5%) [18.5–32.5%]	5 (3.3%) [0.4–6.1%]	0 (0.0%) [0.0–0.0%]
Loss of appetite or eating too much during COVID-19 pandemic	114 (74.5%) [67.5–81.5%]	35 (22.9%) [16.1–29.6%]	4 (2.6%) [0.1–5.2%]	0 (0%) [0.0–0.0%]

Date are presented as n (%) [95% CI].

### The impact of the COVID-19 pandemic on the academic performance of medical postgraduates

Of the medical postgraduates who participated in this study, 54.9% felt that the COVID-19 pandemic had a mild impact on their academic studies. 22.2% felt that the COVID-19 pandemic had a moderate impact on their academics. Two people (1.3%) believed that the COVID-19 pandemic had a severe impact on their academics. And 83 (54.3%) had varying degrees of concern that they would not meet graduation standards due to the COVID-19 pandemic (Table 3). Furthermore, after investigating the changes in postgraduate courses during the COVID-19 pandemic, we found that the proportion of offline courses that changed to online courses increased, resulting in a reduction in practical operations and less interaction between teachers and students (Table 4). This may explain postgraduates' concerns about their academic performance. Most of the postgraduates (85.0%) were not seeking psychological counseling during this time (Table 5). However, most had an increased frequency of academic communication with graduate tutors. In addition, most of them paid usual attention to information and news related to COVID-19 (Table 5).

**Table 3:** The impact of the COVID-19 pandemic on medical graduate studies.

**Questionnaire content**	**Absolutely not (0)** **[95% CI]**	**Mild (1)** **[95% CI]**	**Moderate (2)** **[95% CI]**	**Severe (3)** **[95% CI]**
The overall impact on academics	33 (21.6%) [15.0–28.2%]	84 (54.9%) [46.9–62.9%]	34 (22.2%) [15.6–28.9%]	2 (1.3%) [0.0–3.1%]
Are you worried that you will not meet graduation standards?	70 (45.8%) [37.8–53.7%]	68 (44.4%) [36.5–52.4%]	12 (7.8%) [3.5–12.2%]	3 (2.0%) [0.0–4.2%]

Date are presented as n (%) [95% CI].

**Table 4:** The impact of the COVID-19 Pandemic on medical graduate courses.

**Questionnaire content**	**0** **[95% CI]**	**≤30%** **[95% CI]**	**30–60%** **[95% CI]**	**≥60%** **[95% CI]**
Percentage of off-line courses converted to on-line courses	43 (28.1%) [20.9–35.3%]	44 (28.8%) [21.5–36.0%]	40 (26.1%) [19.1–33.2%]	26 (17.0%) [11.0–23.0%]
Percentage of practice opportunities reduced	52 (34.0%) [26.4–41.6%]	67 (43.8%) [35.8–51.7%]	30 (19.6%) [13.2–26.0%]	4 (2.6%) [0.1–5.2%]

Date are presented as n (%) [95% CI].

**Table 5:** The impact of the COVID-19 pandemic on mental state of medical graduates.

**Questionnaire content**	**Absolutely not (0)** **[95% CI]**	**Occasionally (1)** **[95% CI]**	**Often (2)** **[95% CI]**	**Many times (3)** **[95% CI]**
Frequency of psychological counseling	130 (85.0%) [79.2–90.7%]	21 (14.6%) [8.7–20.4%]	2 (1.3%) [0.0–3.1%]	0 (0.0%) [0.0–0.0%]
Frequency of academic communication with graduate tutors	34 (22.2%) [15.6–28.9%]	59 (38.6%) [30.8–46.4%]	52 (34.0%) [26.4–41.6%]	8 (5.2%) [1.7–8.8%]
Usual attention to information and news related to the new coronary virus	7 (4.6%) [1.2–7.9%]	28 (18.3%) [12.1–24.5%]	91 (59.5%) [51.6–67.3%]	27 (17.6%) [11.5–23.8%]

Date are presented as n (%) [95% CI].

### Mental health and academic performance among medical postgraduates with different genders, grades and academic majors

The comparison between genders revealed that females were experiencing significantly more symptoms in mental health measure scores (*P* < 0.05) (Table 6). The females felt more nervous or anxious during the COVID-19 pandemic than the males (mean 0.77 ± 0.61 vs. 0.42 ± 0.54, *P* < 0.01). The research showed that the female postgraduates were more likely to worry too much about various things because of the COVID-19 pandemic than the males (*P* < 0.01). In addition, female postgraduates more commonly felt that worrying could not be stopped or controlled during the COVID-19 pandemic (mean 0.64 ± 0.67 vs. 0.38 ± 0.53, *P* < 0.05). There were no significant differences in depression symptoms between the males and females (Table 6). In addition, the data showed that there were no significant differences in academic performance between males and females (Supplementary Table 1). Similar results regarding anxiety and depression symptoms were found between the master's class and doctoral class (Table 7). The doctoral students found it harder to relax in a COVID-19 environment (mean 0.63 ± 0.74 vs. 0.31 ± 0.50, *P* < 0.01). What's more, the doctoral postgraduates were more worried that they would not meet graduation standards (mean 0.78 ± 0.78 vs. 0.51 ± 0.58, *P* < 0.05) (Table 8). Among the medical postgraduates, there were no significant differences between the surgical students and internal students in either mental health (Supplementary Table 2) or academic performances (Supplementary Table 3).

**Table 6:** Comparison of the mental health taken by gender.

**Parameter**	**Gender**		***P*-value**
	**Male**	**Female**	
Feeling nervous or anxious during COVID-19 pandemic	0.42 ± 0.54	0.77 ± 0.61	0.001**
Worrying cannot be stopped or controlled during COVID-19 pandemic	0.38 ± 0.53	0.64 ± 0.67	0.021*
Are you worrying too much about various things because of the COVID-19 pandemic	0.42 ± 0.54	0.77 ± 0.63	0.001**
It's hard to relax in a COVID-19 environment	0.42 ± 0.64	0.51 ± 0.67	0.374
Become easily irritable during COVID-19 pandemic	0.46 ± 0.58	0.53 ± 0.65	0.592
Fearing that something terrible is about to happen during COVID-19 pandemic	0.36 ± 0.56	0.45 ± 0.62	0.415
Not feeling motivated or having fun doing things during COVID-19 pandemic	0.36 ± 0.60	0.47 ± 0.65	0.328
Feeling down, depressed or hopeless due to COVID-19 pandemic	0.38 ± 0.58	0.49 ± 0.59	0.265
Difficulty falling asleep, restless sleep or more sleep during COVID-19 pandemic	0.30 ± 0.51	0.33 ± 0.55	0.836
Loss of appetite or eating too much during COVID-19 pandemic	0.28 ± 0.50	0.28 ± 0.51	0.947

Date are presented as mean ± SD. Mann-Whitney U test. ^*^P <0.05; ^**^P <0.01.

**Table 7:** Comparison of the mental health taken by grade.

**Parameter**	**Grade**		***P*-value**
	**Master**	**Doctor**	
Feeling nervous or anxious during COVID-19 pandemic	0.60 ± 0.57	0.70 ± 0.64	0.382
Worrying cannot be stopped or controlled during COVID-19 pandemic	0.51 ± 0.65	0.59 ± 0.63	0.359
Are you worrying too much about various things because of the COVID-19 pandemic	0.60 ± 0.60	0.70 ± 0.64	0.357
It's hard to relax in a COVID-19 environment	0.31 ± 0.50	0.63 ± 0.74	0.007**
Become easily irritable during COVID-19 pandemic	0.44 ± 0.63	0.57 ± 0.63	0.167
Fearing that something terrible is about to happen during COVID-19 pandemic	0.41 ± 0.63	0.42 ± 0.59	0.832
Not feeling motivated or having fun doing things during COVID-19 pandemic	0.41 ± 0.63	0.45 ± 0.65	0.784
Feeling down, depressed or hopeless due to COVID-19 pandemic	0.43 ± 0.55	0.47 ± 0.61	0.786
Difficulty falling asleep, restless sleep or more sleep during COVID-19 pandemic	0.27 ± 0.48	0.36 ± 0.58	0.388
Loss of appetite or eating too much during COVID-19 pandemic	0.24 ± 0.46	0.31 ± 0.54	0.458

Date are presented as mean ± SD. Mann-Whitney U test. ^**^P <0.01.

**Table 8:** Comparison of the academic studies taken by grade.

**Parameter**	**Grade**		***P*-value**
	**Master**	**Doctor**	
The overall impact on academics	0.96 ± 0.65	1.10 ± 0.74	0.200
Are you worried that you will not meet graduation standards?	0.51 ± 0.58	0.78 ± 0.78	0.039*

Date are presented as mean ± *SD*. Mann-Whitney *U* test. *P <0.05.

### Multivariable logistic regression of factors influencing mental health and academic studies of medical postgraduates

In the Multivariable logistic regression analysis (Table 9), compared with male postgraduates, the females were experiencing significantly more symptoms in mental health measure scores during the COVID-19 pandemic (*OR* = 3.256, 95% *CI*: 1.596–6.642; *OR* = 2.510, 95% *CI*: 1.223–5.150; *OR* = 3.011, 95% *CI*: 1.481–6.123). The doctoral students (*OR* = 2.628, 95% *CI*: 1.303–5.301) found it harder to relax in a COVID-19 environment. What's more, the doctoral postgraduates were more worried that they would not meet graduation standards (*OR* = 2.019, 95% *CI*: 1.048–3.889) than the master's class.

**Table 9:** Multivariable logistic regression of factors influencing mental health and academic studies.

	**Gender**				**Grade**			
	**B**	**SE**	**P**	**OR (95%CI)**	**B**	**SE**	**P**	**OR (95%CI)**
**Mental health**								
Feeling nervous or anxious during COVID-19 pandemic	1.180	0.364	0.001**	3.256 (1.596–6.642)	0.337	0.343	0.326	1.401 (0.715–2.744)
Worrying cannot be stopped or controlled during COVID-19 pandemic	0.920	0.367	0.012*	2.510 (1.223–5.150)	0.351	0.342	0.304	1.421 (0.727–2.775)
Are you worrying too much about various things because of the COVID-19 pandemic	1.102	0.362	0.002**	3.011 (1.481–6.123)	0.413	0.342	0.227	1.512 (0.773–2.957)
It's hard to relax in a COVID-19 environment	0.360	0.369	0.330	1.433 (0.695–2.955)	0.966	0.358	0.007**	2.628 (1.303–5.301)
Become easily irritable during COVID-19 pandemic	0.194	0.356	0.586	1.214 (0.604–2.440)	0.603	0.343	0.079	1.828 (0.933–3.581)
Fearing that something terrible is about to happen during COVID-19 pandemic	0.335	0.373	0.368	1.399 (0.673–2.905)	0.169	0.352	0.632	1.184 (0.593–2.363)
Not feeling motivated or having fun doing things during COVID-19 pandemic	0.460	0.380	0.227	1.584 (0.752–3.338)	0.209	0.359	0.560	1.232 (0.610–2.488)
Feeling down, depressed or hopeless due to COVID-19 pandemic	0.381	0.369	0.301	1.464 (0.711–3.015)	0.252	0.349	0.471	1.287 (0.649–2.552)
Difficulty falling asleep, restless sleep or more sleep during COVID-19 pandemic	0.126	0.395	0.751	1.134 (0.522–2.461)	0.376	0.382	0.325	1.457 (0.689–3.080)
Loss of appetite or eating too much during COVID-19 pandemic	0.053	0.408	0.898	1.054 (0.474–2.344)	0.402	0.397	0.311	1.494 (0.687–3.251)
**Academic studies**								
The overall impact on academics	0.070	0.342	0.839	1.072 (0.549–2.095)	0.510	0.332	0.125	1.665 (0.868–3.192)
Are you worried that you will not meet graduation standards?	0.328	0.346	0.344	1.388 (0.704–2.735)	0.702	0.334	0.036*	2.019 (1.048–3.889)

Date are presented as Beta ± SE, OR (95% CI). Multivariable logistic regression analysis. *P <0.05; **P <0.01.

## Discussion

This study used a web-based survey to investigate the mental health and academic performance of medical postgraduates during the COVID-19 pandemic in China. Our major findings are summarized below. First, most of the medical postgraduates had mild anxiety or depression symptoms during the COVID-19 pandemic. Females experienced significantly more symptoms in mental health measure scores than males did. None had a severe depression disorder. Second, 78.43% of participants felt that the COVID-19 pandemic had varying degrees of impact on academics. Moreover, the doctoral postgraduates showed greater academic stress, and they were more worried about not meeting graduation standards than the master's postgraduates.

The outbreak of COVID-19 in the world has had direct and indirect impacts on all areas of society, and the COVID-19 pandemic has lasted for 2 years. Various studies have demonstrated that during the COVID-19 pandemic, mental health problems such as fear, anxiety and depression were common among the general public. A cross-sectional multi-country comparison study demonstrated that substantial variations exist in anxiety and depression symptoms across countries during the COVID-19 lockdown, with personal COVID-19 exposure being a significant risk factor (27). College students who received a large amount of negative information about COVID-19 may be at a greater risk of psychological maladjustment (28–30). Medical students in China face severe depression and anxiety because of their difficult circumstances, such as the long length of schooling, academic pressure, and the stress of clinical practice (31, 32). Therefore, it is of great importance to pay special attention to the psychological status of medical students and to take appropriate interventions to improve their mental health. In the early stages of this pandemic, medical postgraduate education was disrupted and transformed into prolonged home isolation and online learning. People knew little about the novel coronavirus, leading to increased anxiety and depression symptoms in medical students (33, 34). In the later stages, pandemic prevention and control work has become a norm (35). The present study showed that most of the medical postgraduates had mild anxiety or depression symptoms during the COVID-19 pandemic. None had a severe depression disorder. This indicated that most medical students had strong psychological adjustment abilities and adaptability during the pandemic period, which is consistent with previous studies (18, 36). Another reason for this might be the enhanced prevention and control behaviors of postgraduates. The interdisciplinary faculty similarly launched COVID-19 courses focusing on the pathophysiology, diagnosis and treatment of the infection, the health disparities and ethical considerations associated with the pandemic worldwide (37). During the COVID-19 pandemic, especially in the stage of the normalization of pandemic prevention and control work in China, postgraduates have had a relatively positive attitude, doing a good job of protecting themselves in their clinical practice (38).

According to our findings, females experienced significantly more symptoms on mental health measure scores than males did. Previous studies associating gender and mental health have been inconclusive. In a study by Xie et al. (39) males showed more depressive symptoms. However, some previous studies have reported no significant differences in gender with regard to anxiety and depression. This indicates that male and female students experienced similar stresses and negative emotions as a result of the COVID-19 pandemic (29, 40). Another study showed that depression was more prevalent in female students than male students (41). Moreover, a systemic review found that female students experienced higher levels of anxiety and stress (42). These studies are consistent with ours. A possible explanation is that women are more likely to be affected by the COVID-19 pandemic, displaying higher levels of insomnia, sleep disturbances, anxiety and depression (12) and are more likely to report experiencing higher levels of anxiety (43).

The present study also demonstrated that most postgraduates felt that the COVID-19 pandemic had varying degrees of impact on their academics. After investigating the changes in postgraduate courses during the COVID-19 pandemic, we found that the proportion of offline courses changed to online courses has increased, resulting in a reduction in practical operations and less interaction between teachers and students (Table 4). Several barriers to online learning have contributed to students' overall negative experiences. Clinical skills sessions may occur online. Examinations have also been transitioned to online or, in some cases, may be deferred. Many students have reported concerns about not being able to learn and practice clinical skills in person (44). Moreover, there is a decrease in the number of clinical teachers because some of them have devoted their time to pandemic prevention and control teams (45). These factors may explain postgraduates' concerns about their academic performance. Furthermore, the doctoral postgraduates showed greater academic stress, and they were more worried about not meeting graduation standards than the master's postgraduates. This is consistent with a previous study, which indicated that for more senior students, the academic pressure is greater, and some of them face graduation, employment, and practice, etc., but the epidemic of COVID-19 inevitably affects the development of various things (29). In our study, 68.63% of the postgraduates expressed their willingness to participate in volunteer work. Postgraduates have participated in many ways to care for patients and communities in this crisis.

In the midst of this COVID-19 crisis, it is necessary and important to pay special attention to the psychological status of medical students and take appropriate interventions to improve their mental health. In addition, the COVID-19 pandemic may represent an enduring transformation in medical education. There is uncertainty regarding how long this situation will persist and increasing recognition that there may be periods of recurrence in the future. We need more active curricular innovation and transformation to maintain and improve medical students' academic performance. This may be a seminal moment for many disciplines in medicine.

Our study certainly has limitations. First, the sample size was relatively small, but these participants comprised the vast majority of medical postgraduates at the institution in question during the 2 year COVID-19 pandemic. We will combine multiple medical schools distributed over the province to carry out a large sample survey. Additionally, there were a few related factors affecting the postgraduates' mental health and academic performance in this questionnaire. Our follow-up research should strengthen the investigation of related influencing factors.

## Conclusions

Our study found that the COVID-19 pandemic has had a mild impact on the mental health and academic performance of medical postgraduates in China. Females experienced significantly more symptoms on mental health measure scores than males. Moreover, doctoral postgraduates showed greater academic stress, and they were more worried about not meeting graduation standards than master's postgraduates. It is necessary and important to take appropriate interventions to lighten their psychological burdens and improve their academic performance.

## Data availability statement

The raw data supporting the conclusions of this article will be made available by the authors, without undue reservation.

## Ethics statement

The studies involving human participants were reviewed and approved by the Ethics Committee of The Second Affiliated Hospital, School of Medicine, Zhejiang University. The participants provided their written informed consent to participate in this study.

## Author contributions

YY and QT: conceptualization, formal analysis, funding acquisition, writing and preparation of manuscript, and writing original draft. HS, TC, and KY: data curation. YY, YW, and HH: methodology. All authors contributed to the article and approved the submitted version.

## Funding

This study was funded by Zhejiang Provincial Natural Science Foundation of China [LQ20H120009] and The National Natural Science Foundation [Nos. 82101098, 82070939, and 81971667].

## Conflict of interest

The authors declare that the research was conducted in the absence of any commercial or financial relationships that could be construed as a potential conflict of interest.

## Publisher's note

All claims expressed in this article are solely those of the authors and do not necessarily represent those of their affiliated organizations, or those of the publisher, the editors and the reviewers. Any product that may be evaluated in this article, or claim that may be made by its manufacturer, is not guaranteed or endorsed by the publisher.
