# Supplementary material for: The impact of the COVID-19 pandemic on the mental health and academic performance of medical postgraduates
Source: Front Public Health. 2022 Sep 15;10:948710. doi: 10.3389/fpubh.2022.948710 (PMC9521680; doi:10.3389/fpubh.2022.948710)
Supplement: Supplementary file 1 [file Table_1.DOCX]

***Supplementary Material***

**Supplementary Tables**

**Table S1. Comparison of the academic studies taken by gender.**

| **Parameter** | **Gender** | | **P** |
| --- | --- | --- | --- |
|  | **Male** | **Female** |  |
| **The overall impact on academics** | **1.04** $\boldsymbol{\pm}$ **0.81** | **1.02** $\boldsymbol{\pm}$ **0.65** | **0.969** |
| **Are you worried that you will not meet graduation standards?** | **0.62** $\boldsymbol{\pm}$ **0.73** | **0.68** $\boldsymbol{\pm}$ **0.70** | **0.530** |

**Date are presented as *mean*** $\boldsymbol{\pm}$ ***SD*.**

**Mann-Whitney *U* test.**

**Table S2. Comparison of the mental health taken by academic major.**

| **Parameter** | **Academic Major** | | **P** |
| --- | --- | --- | --- |
|  | **Surgical** | **Internal medicine** |  |
| **Feeling nervous or anxious during COVID-19 pandemic** | **0.64** $\boldsymbol{\pm}$ **0.61** | **0.68** $\boldsymbol{\pm}$ **0.61** | **0.658** |
| **Worrying cannot be stopped or controlled during COVID-19 pandemic** | **0.60** $\boldsymbol{\pm}$ **0.68** | **0.50** $\boldsymbol{\pm}$ **0.59** | **0.432** |
| **Are you worrying too much about various things because of the COVID-19 pandemic** | **0.62** $\boldsymbol{\pm}$ **0.60** | **0.69** $\boldsymbol{\pm}$ **0.65** | **0.577** |
| **It’s hard to relax in a COVID-19 environment** | **0.48** $\boldsymbol{\pm}$ **0.65** | **0.49** $\boldsymbol{\pm}$ **0.68** | **0.924** |
| **Become easily irritable during COVID-19 pandemic** | **0.52** $\boldsymbol{\pm}$ **0.61** | **0.50** $\boldsymbol{\pm}$ **0.66** | **0.691** |
| **Fearing that something terrible is about to happen during COVID-19 pandemic** | **0.46** $\boldsymbol{\pm}$ **0.65** | **0.37** $\boldsymbol{\pm}$ **0.54** | **0.460** |
| **Not feeling motivated or having fun doing things during COVID-19 pandemic** | **0.51** $\boldsymbol{\pm}$ **0.67** | **0.34** $\boldsymbol{\pm}$ **0.59** | **0.090** |
| **Feeling down, depressed or hopeless due to COVID-19 pandemic** | **0.46** $\boldsymbol{\pm}$ **0.59** | **0.44** $\boldsymbol{\pm}$ **0.58** | **0.851** |
| **Difficulty falling asleep, restless sleep or more sleep during COVID-19 pandemic** | **0.35** $\boldsymbol{\pm}$ **0.55** | **0.28** $\boldsymbol{\pm}$ **0.51** | **0.366** |
| **Loss of appetite or eating too much during COVID-19 pandemic** | **0.33** $\boldsymbol{\pm}$ **0.54** | **0.22** $\boldsymbol{\pm}$ **0.45** | **0.202** |

**Date are presented as *mean*** $\boldsymbol{\pm}$ ***SD*.**

**Mann-Whitney *U* test.**

**Table S3. Comparison of the academic studies taken by academic major.**

| **Parameter** | **Academic Major** | | **P** |
| --- | --- | --- | --- |
|  | **Surgical** | **Internal medicine** |  |
| **The overall impact on academics** | **1.08** $\boldsymbol{\pm}$ **0.68** | **0.97** $\boldsymbol{\pm}$ **0.73** | **0.232** |
| **Are you worried that you will not meet graduation standards?** | **0.69** $\boldsymbol{\pm}$ **0.72** | **0.62** $\boldsymbol{\pm}$ **0.69** | **0.508** |

**Date are presented as *mean*** $\boldsymbol{\pm}$ ***SD*.**

**Mann-Whitney *U* test.**
